# Evaluation of Clinicopathological Data, the Specific Feline Pancreatic Lipase Assay, and Abdominal Ultrasound as Severity Determinants in Cats with Pancreatitis

**DOI:** 10.3390/vetsci10030209

**Published:** 2023-03-10

**Authors:** Christy Buckley, Alison M. Lee, Robert W. Wills, Alyssa M. Sullivant, Harry Cridge

**Affiliations:** 1 Department of Veterinary Clinical Sciences, College of Veterinary Medicine, Purdue University, West Lafayette, IN 47907, USA; 2 Department of Clinical Sciences, College of Veterinary Medicine, Mississippi State University, Starkville, MS 39762, USA; 3 Department of Basic Sciences, College of Veterinary Medicine, Mississippi State University, Starkville, MS 39762, USA; 4 Department of Small Animal Clinical Sciences, College of Veterinary Medicine, Michigan State University, East Lansing, MI 48824, USA

**Keywords:** Spec fPL, diagnostic imaging, clinical severity

## Abstract

**Simple Summary:**

Pancreatitis is a common inflammatory disease of the exocrine pancreas in cats. Limited data exist to predict the severity of disease. In this study, the medical records of 45 cats with suspected pancreatitis (SP) were used to identify disease severity markers. Selected bloodwork abnormalities, including a high pancreatic lipase concentration, a high total bilirubin concentration, and a low calcium concentration were identified as possible predictors of prolonged hospitalization. Cats with SP that have concurrent gallbladder and stomach abnormalities on abdominal ultrasound (AUS) may require prolonged hospitalization. These findings may help veterinarians identify cats that have more severe disease and require prolonged hospitalization.

**Abstract:**

Limited data exist to predict the severity of pancreatitis in cats. In this retrospective case series, we reviewed the medical records of 45 cats with SP from June 2014 to June 2019. Case definition was based on an internist’s review of clinopathologic data, Spec fPL concentration, and AUS findings. Information extracted from the medical records included signalment, history, physical examination findings, selected clinicopathological data (total bilirubin, glucose, ALP, ALT, and total calcium), Spec fPL concentration, AUS images/clips, length of hospitalization, and survival data. Hazard ratios were used to evaluate the association between clinicopathological data, the Spec fPL assay, AUS findings, and the length of hospitalization. Clinicopathological abnormalities, the Spec fPL, and AUS abnormalities were not statistically associated with the length of hospitalization. Despite a lack of statistical significance, the hazard ratios suggest the potential that an elevated total bilirubin (hazard ratio (HR): 1.19), hypocalcemia (HR: 1.49), and an elevated Spec fPL concentration (HR: 1.54) could be associated with prolonged hospitalization, although additional studies would be needed to verify this. Additionally, hazard ratios suggest that AUS evidence of concurrent gallbladder (HR: 1.61) and gastric abnormalities (HR: 1.36) could be associated with prolonged hospitalization.

## 1. Introduction

Histopathology is considered the gold standard for the diagnosis of acute and chronic pancreatitis in cats; however, it has significant limitations, and it is infrequently used ante-mortem [1,2,3,4]. Given this, many clinicians utilize a clinical diagnosis of pancreatitis composed of an overall assessment of clinical signs, history, bloodwork abnormalities, specific pancreatic lipase concentration, and abdominal imaging findings in each animal [2,3,5,6,7]. 

Patient data, bloodwork, and imaging abnormalities have all been investigated in various populations as potential severity determinants/prognostic indicators. Retrospective studies have identified a history of weight loss, lower body weight at presentation, lethargy, persistent anorexia during hospitalization, pleural effusion, dyspnea, parenteral nutrition, hypocalcemia, hyperkalemia, hypoglycemia, azotemia, an elevated Spec fPL concentration, and an absence of antimicrobial therapy as negative prognostic/severity indicators in cats with pancreatitis [2,8,9,10,11]. With regard to diagnostic imaging, a recent retrospective study in Germany determined that the presence and severity of pancreatic ultrasound abnormalities were not associated with outcome in cats with pancreatitis [12]. Pancreatitis in cats is commonly associated with chronic enteropathy and cholangitis, suspected to be related to the anatomical configuration of the major pancreatic duct and duodenal papilla in cats [3,7]. Concurrent hepatobiliary, gastric, and duodenal abnormalities may also be visualized on abdominal ultrasound. To the authors’ knowledge, the prognostic effect of concurrent ultrasonographic evidence of hepatobiliary and gastric disease has not previously been investigated. 

There were two primary aims of this study. The first aim was to determine if clinicopathological data, the Spec fPL assay, and/or ultrasound findings were associated with clinical severity in a population of cats, as determined by the length of hospitalization. The second aim was to determine if concurrent hepatobiliary and intestinal abnormalities on abdominal ultrasound were associated with the length of hospitalization. 

## 2. Materials and Methods

### 2.1. Case Selection and Data Collection 

Medical records were searched at Mississippi State University between June 2014 and June 2019 for cats that had clinical signs of pancreatitis in addition to having an abdominal ultrasound, serum chemistry, and Spec fPL assay performed within 24 h of each other. These medical records were then reviewed by a board-certified internist (A.M.S.), and each case was defined as suspect pancreatitis (SP) or non-pancreatic disease based on a complete record review. Only those cases diagnosed with SP were included in the study. For cats with multiple hospitalizations, statistical analysis was only performed on the data from the first visit.

### 2.2. Spec fPL Assay

All cats enrolled in the study had serum submitted to a commercial laboratory (Texas A&M University, Gastrointestinal Laboratory, College Station, TX, USA) for the assessment of feline pancreatic lipase immunoreactivity (Spec fPL). The Spec fPL assay has a sensitivity of 54% in mild disease and up to 100% in moderate to severe pancreatitis [13]. The reference interval for Spec fPL is 0–3.5 μg/L. An elevated Spec fPL concentration is highly specific for a diagnosis of pancreatitis in cats [13].

### 2.3. Selected Clinicopathologic Data (tBili, Glucose, ALP, ALT, and tCa)

The serum chemistry values were extracted from the medical records for the following analytes: total bilirubin (Tbili), alkaline phosphatase (ALP), alanine aminotransferase (ALT), blood glucose (BG), and total calcium (tCa). Each value was categorized into the following categories: within the reference interval, below the reference interval, or above the reference interval based on the reference intervals provided by the clinicopathology laboratory at Mississippi State University. Calcium and BG were evaluated as they are commonly referenced prognostic indicators in feline pancreatitis. The total calcium was utilized as ionized calcium was infrequently available when evaluating the medical records. Total bilirubin, ALP, and ALT were utilized to evaluate for potential associations between concurrent hepatobiliary disease and pancreatitis. Other biochemical indicators were not evaluated in this study.

### 2.4. Ultrasonographic Assessment 

Abdominal ultrasound examinations were performed by a radiology resident, under the supervision of a board-certified veterinary radiologist, or directly by the board-certified veterinary radiologist. Ultrasonographic images and video clips were then retrospectively evaluated by a single board-certified veterinary radiologist (A.M.L.) and by a diagnostic imaging specialty intern (C.B.) for standardization. When a consensus was not determined, the images were reviewed by both individuals until a consensus was reached. Ultrasonographic still images and video clips were evaluated for pancreatic, hepatobiliary, and intestinal tract changes as discussed below.

### 2.5. Pancreatic Assessment

The pancreas was evaluated for evidence of pancreatic enlargement, abnormal echogenicity, or abnormal echotexture. The peripancreatic mesentery was also assessed. The pancreatic size was measured in the right limb (<5.9 mm) or body (<9.5 mm) of the pancreas and was compared with previously published reference intervals [14]. The pancreatic echogenicity and echogenicity of the surrounding mesentery were determined by comparison to internal landmarks. The pancreatic echotexture was recorded as normal if the pancreas was homogeneous throughout and was recorded as abnormal if the pancreas was heterogeneous. Pancreatic abnormalities were included in the total ultrasound score (TUS).

### 2.6. Hepatobiliary Assessment

The liver was subjectively categorized as either normal or abnormal based on the assessment of echogenicity relative to internal markers (spleen and renal cortex). Other hepatic abnormalities were noted but were not included in the determination of the TUS. The gallbladder was classified as normal or abnormal based on subjective evaluation of gallbladder sludge, the presence of gallbladder wall edema, or biliary duct dilation (≤4 mm) and objective measurement of the gallbladder wall (<1 mm) and biliary duct thickness (<1 mm), based on previously published reference intervals [15,16]. 

### 2.7. Intestinal Tract Assessment

The stomach was evaluated as abnormal if there was abnormal echogenicity in any layer or if any layer of the of the gastric wall was thickened [17,18,19]. The duodenum was classified as normal or abnormal based on the subjective assessment of the wall layering (abnormal = loss of layering), corrugation, and contractility and the objective assessment based on the wall thickness. Normal duodenal wall thickness was considered <2.4 mm in cats [17].

### 2.8. Total Ultrasound Score

Seven categories of assessment were used to score the ultrasound examinations in these cats. Each category was scored as 0 for normal or as 1 to 2 for abnormal based on the parameters above. See Table 1 for the complete scoring system.

### 2.9. Length of Hospitalization

The hospitalization length was calculated from the date of presentation to the date of discharge and was recorded as an integer.

### 2.10. Statistical Analysis

Cox proportional hazards regression was performed, using the PHREG procedure of SAS for Windows v9.4 (SAS Institute, Inc., Cary, NC, USA), to assess the effect of each of the independent variables, which included TUS, pancreatic ultrasound values, non-pancreatic ultrasound values, selected clinicopathological data, and Spec fPL, on the length of hospitalization in separate models. The assumption of proportionality was tested by determining if the interaction of time and the independent variable was significant in a second model for each of the independent variables. The level of significance was set at an alpha of 0.05.

## 3. Results

### 3.1. Enrolled Animals

Forty-five client-owned (25 male neutered and 20 female spayed) cats were included. The median age of the cats enrolled was 10 years (range, 1 year to 18 years of age). There were 27 domestic shorthair cats, six domestic longhair cats, three Siamese cats, two Himalayan cats, and one each of the following breeds: domestic medium hair, Maine coon, Manx, Persian, Ragdoll, Russian blue, and Snowshoe.

### 3.2. Specific Feline Pancreatic Lipase Assay (Spec fPL)

All cats had a Spec fPL performed. The median Spec fPL concentration was 1.8 μg/L (range, 0.5 μg/L–27.1 μg/L). Seventy-one (32/45) percent of cats had a Spec fPL concentration within the reference interval, whereas 28.9% (13/45) had an elevated Spec fPL concentration. An elevated Spec fPL (HR = 1.54, *p* = 0.44) was not significantly associated with the hospitalization length. The complete data are presented in Table 2.

### 3.3. Selected Clinicopathologic Data (Tbili, ALP, ALT, Blood Glucose, and tCa)

Sixteen percent (7/43) of cases had an elevated total bilirubin, and 84% (36/43) had a normal total bilirubin concentration. The total bilirubin was unavailable for review in two cases. No cats in our study population were hypoglycemic. Eighteen percent (8/43) of cases had an elevated ALP, and 82% (35/43) of cats had a normal ALP. Alkaline phosphatase was unavailable for review in two cases. Thirty percent (13/44) of cases had an elevated ALT, and 70% (31/44) had a normal ALT. Alanine aminotransferase was unavailable for review for one cat. Seven percent (3/43) of cases were hypocalcemic, and 93% (40/43) has a normal total calcium concentration. Elevated total bilirubin (HR = 1.19, *p* = 0.68), elevated ALT (HR = 0.80), *p* = 0.52), elevated ALP (HR = 0.74, *p* = 0.48), and hypocalcemia (HR = 1.49, *p* = 0.52) were not significantly related to the length of hospitalization. The complete data are presented in Table 2.

### 3.4. Ultrasonographic Assessment

#### 3.4.1. Pancreatic Assessment

Thirty one percent (14/45) of cats had an increased pancreatic thickness, whereas 69% (31/45) had a normal pancreatic thickness. Thirty percent (12/45) of cats had a normal pancreatic echogenicity, 48% (22/45) were hypoechoic, and 24% (11/45) had a hyperechoic pancreas. When evaluating the pancreas for echotexture, 78% (35/45) had normal echotexture, and 22% (10/45) of cases had a heterogeneous pancreas. The peripancreatic mesentery was normoechoic in 58% (26/45) of cats, hypoechoic in 2% (1/45), and hyperechoic in 40% (18/45) of cases. The complete data are presented in Table 2.

#### 3.4.2. Hepatobiliary Assessment

Forty-two percent (19/45) of cases had an abnormal hepatic echogenicity, and 58% (26/45) had a normal hepatic echogenicity. The gallbladder was ultrasonographically within normal limits in 69% (31/45) of cases and abnormal in 31% (14/45) of cases. Ultrasonographic evidence of concurrent hepatic (HR = 0.90, *p* = 0.74) and biliary abnormalities (HR = 1.61, *p* = 0.16) was not significantly associated with the length of hospitalization. The complete data are presented in Table 2.

#### 3.4.3. Intestinal Tract Assessment

Eighty-four percent (38/45) of cases were deemed to have a normal duodenum on ultrasound, whereas 16% (7/45) had an ultrasonographically abnormal duodenum. The stomach was ultrasonographically normal in 91% (41/45) of cases and abnormal in 9% (4/45) of cases. Ultrasonographic evidence of concurrent duodenal (HR = 0.85, *p* = 0.70) and gastric abnormalities (HR = 1.36, *p* = 0.57) was not significantly associated with the length of hospitalization. The complete data are presented in Table 2.

#### 3.4.4. Total Ultrasound Score (TUS)

The median TUS was 4 (range, 0–8). Of the ultrasound examinations, 2.2% (1/45) had a TUS of 0, 11.1% (5/45) had a TUS of 1, 17.8% (8/45) had a TUS of 2, 13.3% (6/45) had a TUS of 3, 8.9% (4/45) had a TUS of 4, 8.9% (4/45) had a TUS of 5, 24.4% (11/45) had a TUS of 6, 8.9% (4/45) had a TUS of 7, and 4.4% (2/45) had a TUS of 8. The total ultrasound score was not significantly associated with the length of hospitalization (HR = 1.01, *p* = 0.91). The complete data are presented in Table 2.

#### 3.4.5. Length of Hospitalization

The median length of hospitalization was two days (range, 1–8 days). A right skew was noted.

## 4. Discussion

The ante-mortem diagnosis of pancreatitis in cats is challenging due to the relatively non-specific clinical signs and poor sensitivity and specificity of routine hematology, serum chemistry, and imaging findings [3,20]. Similar to dogs, histopathology is considered the “gold standard” diagnostic test; however, its use is limited by the multi-focal distribution of lesions and potential detection of sub-clinical disease [21]. In fact, a prior study revealed that 45% of apparently healthy cats had evidence of chronic pancreatitis on necropsy, limiting the clinical use of this diagnostic tool [21]. Given the limitations of histopathology, there is a reliance on clinical history, physical examination findings, pancreatic lipase assays and abdominal ultrasonography in the diagnosis and prognostication of pancreatitis in cats. In this retrospective study, we evaluated the association between clinicopathological data, the specific feline pancreatic lipase assay, and abdominal ultrasound findings and the length of hospitalization in a group of forty-five cats. Previously undocumented findings indicate a potential (non-significant) correlation between ultrasonographic evidence of concurrent gallbladder and gastric disease with the length of hospitalization; however, a larger prospective study is required to further investigate this potential association due to the lack of statistical significance noted in this study.

In this study, selected clinicopathological data were evaluated based on prior studies and the aim to investigate whether systemic complications influenced the prognosis in our population of cats. Only a minority of cases in our study (16%) had an elevated total bilirubin concentration. An elevated total bilirubin concentration could be due to compression of the biliary duct from pancreatic enlargement or more likely from concurrent biliary disease. This percentage is lower than previously reported and likely reflects the mild clinical presentation of many cats in this study, with a distinct right skew in the length of hospitalization [2,22,23]. Despite a lack of statistical significance, the hazard ratio (HR: 1.19) could suggest that hyperbilirubinemia was associated with a longer duration of hospitalization (HR: 1.19). Eight cats (18%) had an elevated ALP, and 13 cats (30%) had an elevated ALT. The prevalence of these clinicopathological abnormalities was lower than those reported in the prior literature [2,22,23]. Curiously, the hazard ratios for an elevated ALT (HR: 0.80) and ALP (HR: 0.74) suggest a potential association with a shorter length of hospitalization. The reason for this is unknown; however, evidence of concurrent hepatic abnormalities on bloodwork may prompt the clinician to perform more aggressive supportive therapy, thus decreasing the hospitalization time. A further potential cause is a type II error. No cats in our current study had evidence of hypoglycemia; this is in contrast to the reported literature [2]. Hypoglycemia is a negative prognostic indicator in feline pancreatitis and is suspected to occur as a result of sepsis, liver failure, anorexia, or decreased hepatic glycogen reserves [2]. Our study also investigated the relationship between the total calcium concentration and the length of hospitalization. Only a small portion of cats (7%) in this study were hypocalcemic, which is in contrast with prior studies on pancreatitis [2,10,11]. This may be related to the suspected milder clinical presentation of most cats in this study. Total calcium is also a poor indicator of calcium status in cats [24]. Similar to prior studies, the hazard ratio for hypocalcemia in this study (HR: 1.49) suggests a potential association with a worse outcome/more prolonged hospitalization, but this was not statistically significant [2,10,11].

Specific feline pancreatic lipase immunoreactivity (Spec fPL) is a species-specific immunoassay that measures lipase of pancreatic origin [25]. Given the lack of a commonly accepted gold standard, the sensitivity and specificity of assays for the diagnosis of pancreatitis remain controversial [1]. However, a commonly referenced study documents a sensitivity of 67% and a specificity of 91% for the detection of pancreatitis in cats [13]. A recent study also recommended the use of a SNAP fPL and subsequent fPL, if indicated to rule in or diagnose pancreatitis in cats [23]. In our study, 28.9% of cats had an elevated Spec fPL concentration. This likely corresponds to the milder clinical presentation seen in our study and the over-representation of chronic pancreatitis in cats. In our study, despite a lack of statistical significance, the hazard ratio for an elevated Spec fPL concentration suggests a potential association with an increased length of hospitalization (HR: 1.54). Spec fPL is considered less sensitive in mild disease [13]. Thus, an elevated Spec fPL concentration may represent cats with a more severe disease, which likely requires longer hospitalization. Prior studies have also shown that serum Spec fPL concentrations are prognostic in cats hospitalized with pancreatitis [9].

In our study, we also evaluated the correlation between ultrasonographic abnormalities and the length of hospitalization for suspected pancreatitis. The top three most common pancreatic abnormalities noted in this study were pancreatic hypoechogenicity (48%), hyperechoic peripancreatic mesentery (40%), and pancreatic enlargement (31%). Other abnormalities noted included a hyperechoic pancreas (24%) and a heterogeneous pancreas (22%). A prior study also documented that hyperechoic peripancreatic fat had the highest sensitivity for the detection of pancreatitis, as documented in this study [26]. Historically, the sensitivity of ultrasound for the detection of pancreatitis has been considered low [5,27,28]. However, more recent studies have reported much greater sensitivities for the detection of pancreatitis (62–80%), which may be associated with increases in ultrasound technology [13]. Interestingly a hypoechoic peripancreatic mesentery was noted in one cat, and this had an HR of 1.586. However, given the sample size (one cat), no clinical or statistical inference should be made from this data point. A hyperechoic pancreatic parenchyma was also associated with an HR of 1.307. It is anecdotally reported that a hyperechoic pancreas is associated with chronic pancreatitis in cats. It is possible that chronic disease is associated with a slower resolution of clinical signs and subsequent prolonged hospitalization.

Concurrent chronic enteropathy, cholangiohepatitis, and hepatic lipidosis have been reported in approximately 50% of cats with pancreatitis [5,13,29,30]. It is therefore recommended that the liver and gastrointestinal tract are evaluated at the same time as the pancreas [31]. In our study, 42% of cats had evidence of concurrent hepatic abnormalities on ultrasound, and 31% had evidence of concurrent gallbladder abnormalities. Although not statistically significant, the hazard ratio for concurrent ultrasonographic gallbladder disease suggests an association with the length of hospitalization (HR: 1.61). Future prospective studies would be required to confirm this suspicion. Seven cats (16%) in this study had concurrent evidence of duodenal abnormalities, and four cats (9%) had concurrent gastric abnormalities. Despite a lack of statistical significance, the hazard ratios suggest the potential that concurrent gastric abnormalities on ultrasound could be associated with the length of hospitalization (HR: 1.36). Gastric wall thickening was recently documented in a case series of dogs with pancreatitis, and these data suggest that gastric wall thickening may also be a complication of pancreatitis in cats [32].

Due to the absence of mortality in the population of cats evaluated, the length of hospitalization was used as a surrogate indicator of the severity of pancreatitis. The length of hospitalization as a severity indicator has several limitations as it is affected by numerous factors other than disease severity. Other factors that may affect the length of hospitalization include efficacy of therapy in addition to clinician and owner decision making. Furthermore, the population of cats evaluated in this study represents cats seen at a referral institution. These cats may have spent varying periods of time under the care of the owner or primary care veterinarians before arriving at our facility. This could impact measures of disease severity based on the length of hospitalization. The patient population in our study was right skewed with regard to the length of hospitalization, and this may have contributed to the lack of statistical significance noted in this study.

In this study, cats with SP were diagnosed based on the evaluation of a panel of data including history, physical examination findings, clinicopathological data, Spec fPL concentration, and abdominal ultrasound findings by a board-certified internist (A.M.S). Although combining the results of the tests likely improves the detection of pancreatitis, there is no consensus with regard to the clinical diagnosis of pancreatitis, and as such, slight differences may be noted between different evaluators. A further limitation of our study was the use of total calcium concentration to assess the calcium status. Total calcium has been shown to a poor indicator of ionized calcium in cats, with a 40% diagnostic discordance in the prediction of ionized calcium using total calcium [24]. This likely limited our ability to accurately determine the effect of calcium status on the length of hospitalization in our patient group. In spite of this, the hazard ratio for total hypocalcemia still suggests an association with the length of hospitalization (HR: 1.49), as anticipated based on prior studies [2,10,11]. Ultrasound examinations were performed by different individuals during this study; however, all still images and video clips were subsequently evaluated by two individuals (A.M.L. and C.B.). Pancreatic ultrasonography is highly dependent on the skill of the ultrasonographer, and as such, this may have influenced the results of our study [7]. The effect of the user was minimized in this study by retrospective evaluation of the images by two individuals (A.M.L. and C.B.) and determination of an imaging consensus. We failed to include an assessment of abdominal lymphadenopathy and free abdominal fluid in the score system. Additionally, we only evaluated one portion of the intestinal tract. This may influence the results of this study. Large-scale prospective studies are required to further investigate the relationships outlined in this manuscript.

## 5. Conclusions

Selected bloodwork abnormalities, including a high pancreatic lipase concentration, a high total bilirubin concentration, and a low calcium concentration, were identified as possible predictors of prolonged hospitalization, although the results were not statistically significant. Cats with concurrent gallbladder and stomach abnormalities on abdominal ultrasound (AUS) may require prolonged hospitalization.

## Figures and Tables

**Table 1:** Ultrasonographic findings in 45 cats with suspected pancreatitis.

Assessment	Normal	Abnormal (+1)	Abnormal (+2)
Pancreatic size	31	14	
Pancreatic echogenicity	12	11 **	22 *
Pancreatic echotexture	10	35	
Mesenteric echogenicity	26	1 *	18 **
Liver echogenicity	26	19	
Gallbladder	31	14	
Duodenum	38	7	
Stomach	41	4	

This table represents the total scoring value (number of cats) for each evaluated parameter by ultrasonography. The total number of cats was 45 for each parameter. Parameters with multiple levels of scoring are denoted by * for a hypoechoic change and ** for a hyperechoic change.

**Table 2:** Clinicopathologic, ultrasonographic, and Spec fPL statistical reference data.

Variable	Hospital Stay (*p*-Value)	Hospital Stay (Hazard Ratio)
Elevated Spec fPL	0.4363	1.539
Elevated tBili	0.6835	1.191
Hypoglycemia	N/A	N/A
Elevated ALP	0.4766	0.741
Elevated ALT	0.5269	0.803
Hypocalcemia	0.5188	1.493
Pancreatic size	0.8666	1.058
Pancreatic hyperechogenicity	0.5514	1.307
Pancreatic hypoechogenicity	0.7215	1.147
Pancreatic echotexture	0.6403	0.840
Mesenteric hypoechogenicity	0.6624	1.586
Mesenteric hyperechogenicity	0.7310	0.896
Liver	0.7380	0.900
Gallbladder	0.1607	1.605
Duodenum	0.7047	0.852
Stomach	0.5726	1.360
TUS	0.9140	1.008

This table represents the *p*-values and hazard ratios for each data point collected. A hazard ratio greater than 1 indicates an increased chance of a longer hospital stay.

## Data Availability

Data are only available on request due to restrictions. As the study was retrospective, no owner consent was signed, and personal data are therefore not publicly available.
